# Late Male-Killing Viruses in *Homona magnanima* Identified as Osugoroshi Viruses, Novel Members of *Partitiviridae*

**DOI:** 10.3389/fmicb.2020.620623

**Published:** 2021-01-20

**Authors:** Ryosuke Fujita, Maki N. Inoue, Takumi Takamatsu, Hiroshi Arai, Mayu Nishino, Nobuhiko Abe, Kentaro Itokawa, Madoka Nakai, Syun-ichi Urayama, Yuto Chiba, Michael Amoa-Bosompem, Yasuhisa Kunimi

**Affiliations:** ^1^Laboratory of Sanitary Entomology, Faculty of Agriculture, Kyushu University, Fukuoka, Japan; ^2^Department of Medical Entomology, National Institute of Infectious Diseases, Tokyo, Japan; ^3^Department of Applied Biological Science, Tokyo University of Agriculture and Technology, Fuchu, Japan; ^4^Pathogen Genomics Center, Natinal Institute of Infectious Diseases, Tokyo, Japan; ^5^Department of Life and Environmental Sciences, University of Tsukuba, Tsukuba, Japan

**Keywords:** male killing, *Homona magnanima*, *Partitiviridae*, horizontal transmission, insect virus

## Abstract

Late male-killing, a male-specific death after hatching, is a unique phenotype found in *Homona magnanima*, oriental tea tortrix. The male-killing agent was suspected to be an RNA virus, but details were unknown. We herein successfully isolated and identified the putative male-killing virus as Osugoroshi viruses (OGVs). The three RNA-dependent RNA polymerase genes detected were phylogenetically related to Partitiviridae, a group of segmented double-stranded RNA viruses. Purified dsRNA from a late male-killing strain of *H. magnanima* revealed 24 segments, in addition to the RdRps, with consensus terminal sequences. These segments included the previously found male-killing agents MK1068 (herein OGV-related RNA16) and MK1241 (OGV-related RNA7) RNAs. Ultramicroscopic observation of purified virions, which induced late male-killing in the progeny of injected moths, showed sizes typical of Partitiviridae. Mathematical modeling showed the importance of late male-killing in facilitating horizontal transmission of OGVs in an *H. magnanima* population. This study is the first report on the isolation of partiti-like virus from insects, and one thought to be associated with late male-killing, although the viral genomic contents and combinations in each virus are still unknown.

## Introduction

Maternally inherited female-biased sex ratio is caused by a variety of mechanisms, including male-killing. The phenomenon of male-killing can be categorized into two types: early (male-specific death in the embryonic stage) or late (male-specific death in the larval or pupal stage), both of which result in female-biased sex ratios (Hurst, 1991, 1993; Dunn and Smith, 2001). Male killing in insects has been reported to be induced by infection with bacteria (e.g., with *Wolbachia, Rickettsia, Spiroplasma, Flavobacteria*, or *Arsenophonus*) or microsporidia (Skinner, 1985; Hurst et al., 1999; Huigens et al., 2000; Fukatsu et al., 2001; Morimoto et al., 2001; Agnew et al., 2002; Perlman et al., 2006; Gruwell et al., 2007; Hedges et al., 2008; Werren et al., 2008; Arai et al., 2020). *Homona magnanima*, oriental tea tortrix, is known as a pest for tea plants in East Asia. It also feeds on a variety of plant leaves, including apple trees and citrus. Previously, we identified a male-killing agent, suspected to be an RNA virus, in *H. magnanima* (Nakanishi et al., 2008). The cause of the male-killing was identified as RNA fragments (MK1068 and MK1241); however, the details were not understood.

Viruses in insects are transmitted via two ways, horizontal transmission and vertical transmission. Most insecticidal viruses such as baculovirus spread their progeny by killing host insects and infecting other hosts (horizontal transmission) (Rohrmann, 2019). In this process, insecticidal functions facilitate virus escape from infected host cells and an efficient progeny virus spread. Dengue virus, a member of Flavivirus, is a mosquito-borne virus that infects host mosquitoes via blood-sucking. Dengue virus is also maintained in its lifecycle via vertical transmission (Lequime and Lambrechts, 2014). Dengue virus can be transferred to an egg in an infected female, which enables viral survival regardless of blood-feeding to virus-permissive animals. Thus, the insect viruses choose one of or both transmission methods to survive in the ecological system.

In this study, we identified the previously suggested late male-killing virus as Osugoroshi viruses (OGVs), considered as *Partitiviridae-*related viruses. We also discuss the role of the late male-killing function of OGVs with consideration for their survival strategy.

## Materials and Methods

### Insects

*H. magnanima* (egg masses, larvae, or pupae) were collected at tea plantations in TYO (Tokyo Metropolitan), TKR (Saitama Pref.), AMI (Ibaraki Pref.), KWM (Miyazaki Pref.), KBY (Miyazaki Pref.), UJI (Kyoto Pref.), SMD (Shizuoka Pref.), YMK (Kanagawa Pref.), SZK (Shizuoka Pref.), NNB (Yamanashi Pref.), and MNM (Kagoshima Pref.) in Japan (Supplementary Figure 1). The collected *H. magnanima* populations were individually maintained in the lab (25°C, 16L8D) on a SilkMate 2S artificial diet (Nosan Co., Japan). The larvae of SMD and TYO strains were fed with SilkMate 2S containing 0.1% tetracycline to eliminate bacteria that may have influenced sex ratio [NSR-SMD strain (Tsugeno et al., 2017) and NSR-TYO strain (Arai et al., 2019), respectively]. After the establishment of NSR strains, larvae were fed with SilkMate 2S without tetracycline. The late male-killing strain established from the SMD population was maintained by mating with NSR-SMD males.

### Nucleic Acid Extraction, PCR, and RT-PCR

DNA was isolated from *H. magnanima* pupae or adults as follows. *H. magnanima* was homogenized in lysis buffer (10 mM Tris-HCl pH 8.0, 100 mM EDTA, 1% SDS), then treated with Proteinase K at 50°C for 5 h. The lysates were further incubated with RNase A, and then proteins were removed by using Protein Precipitation Solution (Qiagen). DNA was precipitated with isopropanol and resuspended in distilled water.

RNA in *H. magnanima* was isolated from lysates using Isogen (Nippon Gene) according to the manufacturer's instructions. The extracted RNA was further treated with DNase I to remove DNA contaminants. Complementary DNA was synthesized using AMV Reverse Transcriptase XL (Takara) according to the manufacturer's instructions.

The DNA or cDNA was used as a template for PCR, which was carried out with *Ex*Taq (Takara) using specific primer sets, listed in Supplementary Table 3. Products were separated by agarose gel electrophoresis.

The dsRNA purification was carried out using CF11-cellulose. Briefly, total RNA was extracted as described above, then mixed with CF-11 cellulose in binding buffer (50 mM Tris-HCl pH 6.8, 130 mM NaCl, 1 mM EDTA, 16% ethanol, 5% β-mercaptoethanol). Unbound nucleic acids were washed out twice with binding buffer. The resin was incubated in elution buffer (50 mM Tris-HCl pH 6.8, 130 mM NaCl, 1 mM EDTA) to release dsRNAs. The dsRNAs were recovered by ethanol precipitation and resolved in RNase-free water. The purified dsRNAs were analyzed by 1.5% agarose gel electrophoresis.

### Next-Generation Sequencing Analysis and RACE Sequencing

*H. magnanima* female adults of the male-killing strain (hereinafter referred to as the late strain) were homogenized in PBS as above and passed through a 0.45 μm filter. The filtrates were treated with RNase A and DNase I as described previously (Fujita et al., 2016); then RNA was extracted using Isogen II (Nippon Gene). Construction of a cDNA library and sequencing analysis was carried out according to the method described in Fujita et al. (2016). Reads were analyzed with CLC Genomic Workbench software (CLC bio) or the SPAdes algorithm (Bankevich et al., 2012). Complete OGV genomic sequences were determined by the FLDS sequencing method as described previously (Urayama et al., 2016). Complete OGV nucleotide sequences were submitted to the DDBJ/GenBank/EMBL database under accession numbers LC383810-LC383814 and LC597875-LC597896.

### Sequence Analysis

Phylogenetic analysis using partitivirus RdRp amino acid sequences was carried out as follows. The RdRp sequences were first aligned using ClustalW (*http://clustalw.ddbj.nig.ac.jp/*), then the aligned matrix data were confirmed manually. The NCBI accession numbers of the sequences used in this analysis are listed in Supplementary Table 4. The amino acid sequences completely conserved among viruses were analyzed in MEGA7 (Kumar et al., 2016) using the maximum likelihood method with the JTT matrix model. The statistical significance of the resulting tree was evaluated using a bootstrap test with 1,000 replications.

### Virion Purification and Electron Microscopy

*H. magnanima* late strain adult females were homogenized in PBS, and large debris was removed by centrifugation. Virions in the homogenates were separated by ultracentrifugation (100,000 × g, 3 h, 4°C) with 20 and 50% sucrose cushions. The virion-containing layer was recovered and further separated in a 20–50% sucrose gradient solution (100,000 × g, 16 h, 4°C). The recovered virions were dialyzed using 10 mM Tris-HCl buffer (pH 7.4) and then observed by electron microscopy.

The purified virions were loaded on membrane-coated transmission electron microscopy grids and stained with 2% phosphotungstic acid solution (pH 7.0). The samples were observed using a JEM-2100 transmission electron microscope (Jeol).

## Results

We previously identified two RNA fragments as the components of the late male-killing agent for *H. magnanima*. Because those two RNAs did not show any specific similarity with known cellular genes, we speculated that the male-killing agent is an RNA virus. To isolate and identify the virus, we first collected 18 *H. magnanima* populations from 10 regions (Supplementary Figure 1) and examined the sex ratio of their progeny. RT-PCR targeting MK1241 RNA showed that 12 of the 18 populations (97/636 tested individuals in total) carried the putative late male-killing virus, although most of the tested populations showed typical sex ratios (Table 1). Then, we cultured the collected *H. magnanima* SMD strain (see Methods and Supplementary Figure 1) under laboratory condition. *Wolbachia* was eliminated from an SMD strain by feeding with an artificial diet containing tetracycline. The resultant *H. magnanima* strain exhibiting late male-killing was designated as the late strain. This late strain carried MK1241 RNA but was free from *Wolbachia* and *Spiroplasma* infection (Supplementary Figure 2). We also established a strain from the SMD *H. magnanima* population, which was infected with neither MK1241 RNA, *Spiroplasma*, nor *Wolbachia* (Supplementary Figure 2). The progeny of this strain showed a normal sex ratio and is hereinafter referred to as the non-biased sex ratio (NSR-SMD) strain. Because all late strain males died before the adult stage, the late strain was maintained by mating with NSR-SMD males. We also determined hatchability and mortality for each strain. The hatchability of the late strain was almost the same as that of the NSR-SMD strain, but 68% of late strain hatchlings died before adult emergence (Supplementary Figure 2).

**Table 1:** The sex ratios and prevalence of MK1241 RNA in natural populations of *Homona magnanima* collected from tea fields in Japan.

**Year**	**Month**	**Population^a^**	**No. of collected samples at each stage**							**No. of pupae examined**		**Pupal sex ratio (% male)**	**No. of adults examined**		**Adult sex ratio (% male)**	**% adult prevalence rate of MK1241 RNA (No. of tested sample)**
			**Egg**	**Larvae**					**Pupa**	**Female**	**Male**		**Female**	**Male**		
				**1st**	**2nd**	**3rd**	**4th**	**5th**								
2007	Mar	TKR	0	0	0	2	2	19	0	11	8	0.42	6	8	0.57	30.0 (10)
	Mar	AMI	0	0	0	33	30	30	0	31	17	0.35	28	16	0.36	41.9 (31)
	May	KWM	0	0	0	0	3	82	9	47	30	0.39	37	24	0.39	14.9 (47)
	May	KBY	0	0	0	0	6	44	1	22	24	0.52	20	24	0.55	0.0 (23)
	Jun	TKR	0	2	1	4	24	14	0	14	19	0.58	12	15	0.56	15.4 (13)
	Jun	AMI	0	0	0	8	41	46	0	33	16	0.33	23	9	0.28	37.5 (32)
	Jun	UJI	0	0	1	9	7	0	0	7	8	0.53	6	8	0.57	0.0 (7)
	Sep	SMD	98	0	0	0	0	0	0	1239	1072	0.46	1041	1000	0.49	7.1 (42)
2008	Apr	TKR	0	0	0	11	46	122	0	78	67	0.46	61	56	0.48	25.7 (74)
	Jun	TKR	0	0	0	1	46	44	0	41	26	0.39	35	22	0.39	21.1 (38)
	Jun	YMK	0	0	0	0	27	47	0	33	25	0.43	22	19	0.46	23.5 (34)
	Jul	TKR	0	2	11	31	67	94	2	74	69	0.48	58	59	0.50	18.3 (71)
	Jul	SMD	0	0	0	2	23	43	0	32	27	0.46	27	26	0.49	0.0 (28)
	Aug	SZK	0	0	0	7	17	35	0	22	18	0.45	12	15	0.56	0.0 (21)
	Aug	NNB	0	0	0	0	7	28	0	16	13	0.45	13	8	0.38	0.0 (16)
	Sep	TKR	0	0	0	8	40	48	0	31	26	0.46	26	26	0.50	0.0 (30)
	Nov	MNM	7	0	0	0	0	55	86	56	51	0.48	28	33	0.54	6.2 (65)
	Dec	TKR	4	6	53	25	52	25	0	52	66	0.56	39	55	0.59	9.3 (54)

a *For the abbreviations, see Supplementary Figure 1*.

To identify the virus in the late strain, we carried out next-generation sequencing (NGS) analysis. For the extraction of viral RNA, late strain female moths were homogenized and centrifuged at low speed. The supernatant was treated with DNase and RNase to concentrate viral RNAs (Fujita et al., 2016). Total RNA was isolated from the nuclease-treated homogenates and used as a template for cDNA library construction. In the NGS analysis, we obtained 27 contigs (Supplementary Table 1), and as expected, sequences consistent with MK1241 and MK1068 sequences were included in the contigs (MK-3 and MK-14, respectively). We successfully identified two contigs encoding RNA-dependent RNA polymerases (RdRp) related to *Partitiviridae* viruses (MK-11 and MK-25). Another assembler algorithm, SPADes (Bankevich et al., 2012), detected one more partitiviral RdRp gene (MKsp30) (Supplementary Table 2). Thus, late strain female moths carried at least three novel partiti-like RdRp sequences.

**Figure 1:**
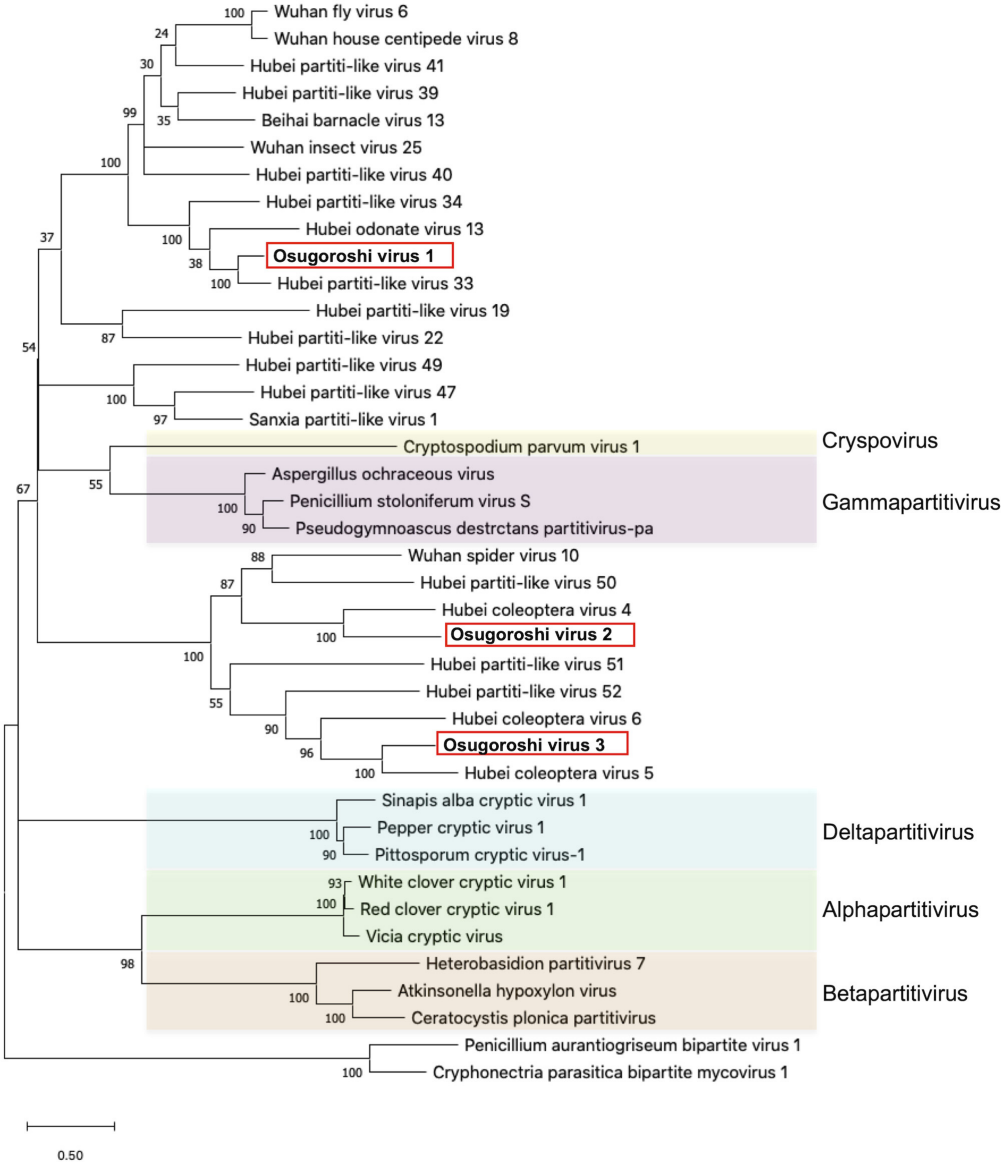
Identification of partitiviruses in the *Homona magnanima* late strain. Phylogenetic analysis of the amino acid sequences of partitivirus RdRp was represented. The top 10 hits from BLAST analysis of Osugoroshi virus (OGV) 1, OGV2 (MK-25), and OGV3-encoded RdRp and 13 viruses categorized in previously defined genera (colored) were used in the alignment of two outgroup viruses (see also Supplementary Table 4).

The putative partitiviral RdRp sequences (MK-11, MK-25, and MKsp30) were significantly different from each other, but all contained conserved motifs (Supplementary Figure 3) (Vásquez-Del Carpio et al., 2006), suggesting that these RdRps are active RNA polymerases. Blast analysis revealed MK-11, MK-25, and MKsp30 to be novel, unclassified *Partitiviridae* species (King et al., 2011). We designated these three partitiviruses Osugoroshi virus as (OGV) 1, 2, and 3, respectively (Figure 1). In the phylogenetic tree, OGV2 and OGV3 were located in the same clade and showed similarity to Hubei coleoptera virus 5 and Hubei coleoptera virus 4, respectively (Shi et al., 2016). Because all viral-like sequences in this clade were found from Coleoptera or Araneae, these viruses (or viral-like sequences) are thought to be invertebrate-specific. OGV1 was located in a different clade and showed similarity to Hubei partiti-like virus 33, which also appeared in Coleoptera. Based on this data, OGVs and other related viruses were considered a group of invertebrate partitiviruses.

**Figure 2:**
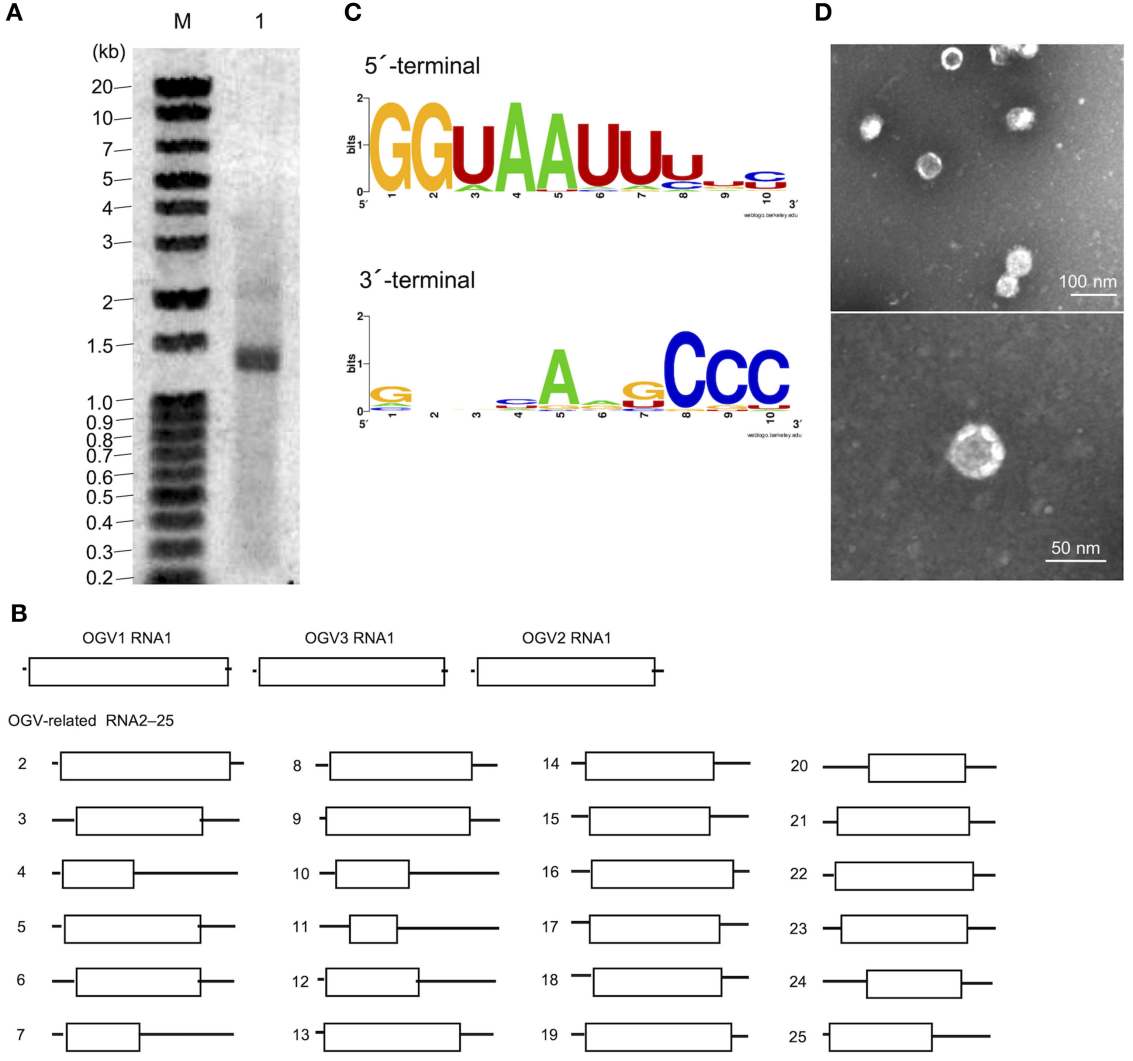
Identification of Osugoroshi viruses (OGVs) in the *Homona magnanima* late strain. **(A)** dsRNA was isolated from the *H. magnanima* adult females of the late strain (lane 1) and separated by 1.5% agarose gel electrophoresis. The DNA marker was also shown (lane M). **(B)** The schematics of the identified OGVs' RNA1 and other OGV-related RNAs. Boxes indicated the position of open reading frames (ORFs). **(C)** The consensus sequences of the first and the last ten nucleotides among the 27 identified OGV's RNAs (in sense strands). **(D)** Electron microscopic image of viral particles purified from the adult female's homogenate of the late strain. The particles were negatively stained, and the scale was indicated in each panel.

From this data, we assumed that OGVs are partiti-like viruses with a segmented dsRNA genome. Therefore, we purified dsRNA from the *H. magnanima* late strain for detailed analysis. The purified dsRNAs was about 1.3 kb in size (Figure 2A). In the primary NGS virome analysis, we found 20 other contigs uniquely appearing in the late strain, some of which were suspected to be components of OGVs (Supplementary Table 2 and Supplementary Figure 4). To verify this, we carried out complete sequencing of the purified dsRNAs with the FLDS method. We successfully determined the sequences of 27 contigs, which included OGV1–3 RNA1, MK1068, and MK1241 (Table 2 and Figure 2B). The length of the 27 RNAs ranged between 1,200 and 1,497 bp, corresponding to the agarose gel electrophoresis data (Table 2 and Figure 2A). Interestingly, they all had consensus in their 5′- and 3′-terminal sequences (GGUAAUU on 5′-terminus and ANG/UCCC on 3′-terminus, in sense strands). OGV1 RNA1 had complete consensus in the first seven nucleotide sequences with OGV-related RNA2–25, with the exception of RNA16. OGV2 RNA1 had different sequences on its 5′-termini (GGAAACA) and 3′-termini (ACCCGT), and they were almost the same as OGV-related RNA16 (5′: GGAAAUA, 3′: ACCCGT). The 5′-terminal sequence of OGV3 RNA1, GGAAUAG, was partly similar to the 5′-terminal sequence of OGV1 RNA1. On the other hand, the 3′-terminal sequence was somewhat similar to OGV2 RNA1 and OGV-related RNA16. Considering the nucleotide sequence structures and the lack of poly-A tails, we concluded that these dsRNAs were components of the newly identified partiti-like viruses, OGVs.

**Table 2:** Contigs determined in FLDS analysis using purified ds RNA of *Homona magnanima* late strain.

**Contig name**	**Read counts (FLDS)**	**Length (nts)**	**Product**	**Encoded protein (kDa)**	^*^Corresponds to the contig	**The first 10 nucleotides**	**The last 10 nucleotides**
OGV1 RNA1	3,099	1,497	RdRp	54.4	MK11	GGTAATTTAT-	-GGTTAGTCCC
OGV3 RNA1	5,718	1,393	RdRp	49.9	MKsp30	GGAATAGTTC-	-GCACCCCGTA
OGV2 RNA1	1,774	1,382	RdRp	48.6	MK25	GGAAACATTT-	-GGTGACCCGT
OGV-related RNA 2	11,670	1,364	unknown	44.6	MK16	GGTAATTTCG-	-GAGCAAGCCC
OGV-related RNA 3	19,084	1,337	unknown	34.4	MK15	GGTAATTTAC-	-GATTAGGCCC
OGV-related RNA 4	18,356	1,321	unknown	19.6	MK9	GGTAATTTTC-	-CCAAGTTCCC
OGV-related RNA 5	2,847	1,315	unknown	36.7	MK27	GGTAATTTGT-	-GTGCAAGCCC
OGV-related RNA 6	33,872	1,304	unknown	34.3	MK4	GGTAATTTAA-	-GGTTAGGCCC
OGV-related RNA 7	12,147	1,294	unknown	20.8	MK3	GGTAATTTTC-	-CCCAAAGCCC
OGV-related RNA 8	10,020	1,292	unknown	38.0	MK22	GGTAATTTGT-	-GTACAAGCCC
OGV-related RNA 9	15,191	1,291	unknown	38.7	MK10	GGTAATTCGT-	-GTGCAAGCCC
OGV-related RNA 10	14,245	1,290	unknown	20.1	MK5	GGTAATTTCG-	-ACCCAGTCCC
OGV-related RNA 11	2,575	1,283	unknown	12.6	MK20	GGTAATTTTC-	-TGCAGGTCCC
OGV-related RNA 12	5,385	1,282	unknown	25.1	MK13	GGTAATTCGT-	-GAGCAAGCCC
OGV-related RNA 13	13,580	1,279	unknown	36.1	–	GGTAATTTTC-	-GTGCATGCCC
OGV-related RNA 14	338,949	1,277	unknown	34.6	MKsp11	GGTAATTATC-	-GATTAAGCCC
OGV-related RNA 15	8,245	1,271	unknown	32.6	MK17	GGTAATTTTC-	-GGTTAGGCCC
OGV-related RNA 16	7,770	1,269	unknown	37.8	MK14	GGAAATACAT-	-ATGAACCCGT
OGV-related RNA 17	9,601	1,267	unknown	34.6	MK1	GGTAATTTTC-	-ACGCAAGCCC
OGV-related RNA 18	8,235	1,265	unknown	35.1	MK18	GGTAATTATC-	-GGTTAAGCCC
OGV-related RNA 19	3,764	1,261	unknown	39.4	MK19	GGTAATTTTC-	-ACGCAAGCCC
OGV-related RNA 20	12,372	1,239	unknown	26.0	MK23	GGTAATTCGT-	-CACAAGTCCC
OGV-related RNA 21	3,527	1,235	unknown	35.4	MK2	GGTAATTTTC-	-GTGCAAGCCC
OGV-related RNA 22	27,072	1,233	unknown	37.1	MKsp26	GGTAATTCGT-	-GTGCAGTCCC
OGV-related RNA 23	5,817	1,230	unknown	34.9	MK26	GGTAATTTTC-	-GGTTAGGCCC
OGV-related RNA 24	12,872	1,220	unknown	25.7	MK24	GGTAATTCGT-	-CCATAGTCCC
OGV-related RNA 25	4,246	1,200	unknown	26.9	MK12	GGTAATTTTC-	-GTGCAAGCCC

* *See Supplementary Tables 1, 2*.

We then tried to purify the OGV virions from homogenates of *H. magnanima* late strain. The fractionation of virions in sucrose gradient ultracentrifugation led to the successful isolation of viral particles with an estimated size of 30 nm (Figure 2D). SDS-PAGE analysis of the purified fraction revealed a relatively major band around the 29 kDa molecular size mark; however, the crude composition of proteins in the sample made it difficult to identify structural proteins (Supplementary Figure 5). All the dsRNAs carried at least one open reading frame with estimated product sizes between 12.6–54.4 kDa; however, the lack of significant similarity to known genes prevented accurate prediction of their function(s) (Figure 2B and Table 2). Alignment with reported partitiviral capsid proteins (CPs) and phylogenetic analysis did not show a clear cluster, and failed to identify OGVs' CP genes. This observation may be due to the low similarity between the sequences and genomic reassortment between partitiviruses, facilitated by the encapsidation of partitivirus genomic RNA (RNA 1 and 2) in separate capsids (Nibert et al., 2013).

**Figure 3:**
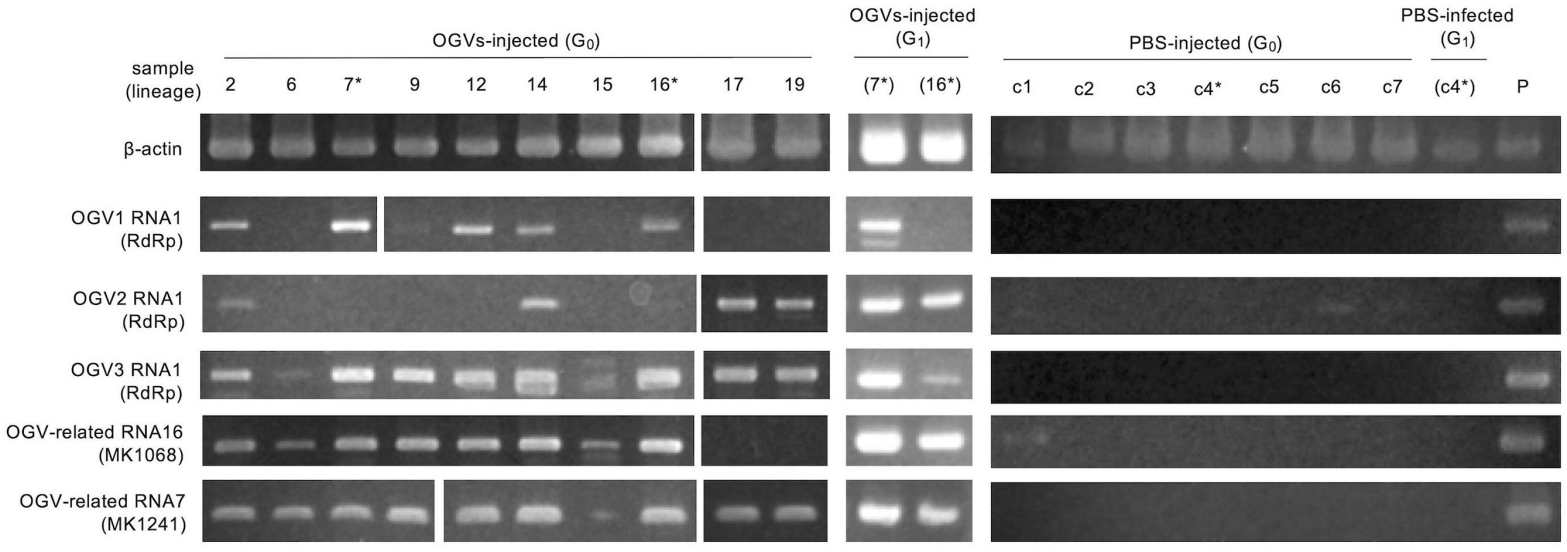
Injection of purified Osugoroshi virus (OGV) virions into the *Homona magnanima* non-biased sex ratio (NSR) strain. RT-PCR confirmed OGV infection. Fourth instar larvae of *H. magnanima* NSR-TYO strain were injected with purified OGVs or phosphate-buffered saline (PBS), and total RNA was extracted from 10 adult female moths (G_0_). G_1_ adult females were generated from the G_0_ females (asterisks) by mating with NSR males. The total RNA from adult females of the laboratory-maintained late strain was used as a positive control (P). *shows the linage relationships.

To determine whether OGVs are responsible for late male-killing, we inoculated purified viral particles (Figure 2C) into 17 NSR-TYO 4th-instar larvae and traced OGVs' RNA1 and OGV-related RNA7 (MK1241) and 16 (MK1068), the previously identified markers for male-killing agents (Nakanishi et al., 2008). Ten adult female moths (samples 2, 6, 7, 9, 12, 14, 15, 16, 17, and 19 in Figure 3) were obtained in G_0_ and mated with NSR-TYO males. OGV infection status was examined by RT-PCR using RNA extracted from adult females after oviposition. OGV3 RNA1 and OGV-related RNA7 appeared in most G_0_ individuals (Figure 3). On the other hand, OGV1 RNA1, OGV2 RNA1, and OGV-related RNA16 were found only in five, four, and eight individuals, respectively. From these moths, two lineages were obtained (lineage 7 and 16) in the next generation (G_1_), both of which exhibited a female-biased sex ratio due to late male-killing (Numbers of male and female moths are, respectively, 0 and 31 in lineage 7, and 0 and 3 in lineage 16). An adult female was selected from each lineage and checked for OGV infection. Moths in lineage 7 possessed all the five tested RNAs. In the G_1_ adult female lineage 16, all but OGV1 RNA1 were detected, although it had appeared in the maternal moth. Despite the absence of OGV1 RNA1, G_1_ of lineage 16 exhibited complete late male-killing, suggesting that OGV1 RNA1 may not be necessary for late male-killing in *H. magnanima*. In the control group, phosphate-buffered saline (PBS) was injected into 68 NSR-TYO 4th-instar larvae, and 13 female moths oviposited. The percentage of females in G_1_ (sample c4 in Figure 3) was 42.9%, and no OGV RNAs were detected in this group. Based on these results, we conclude that OGV was responsible for inducing late male-killing in *H. magnanima*.

**Figure 4:**
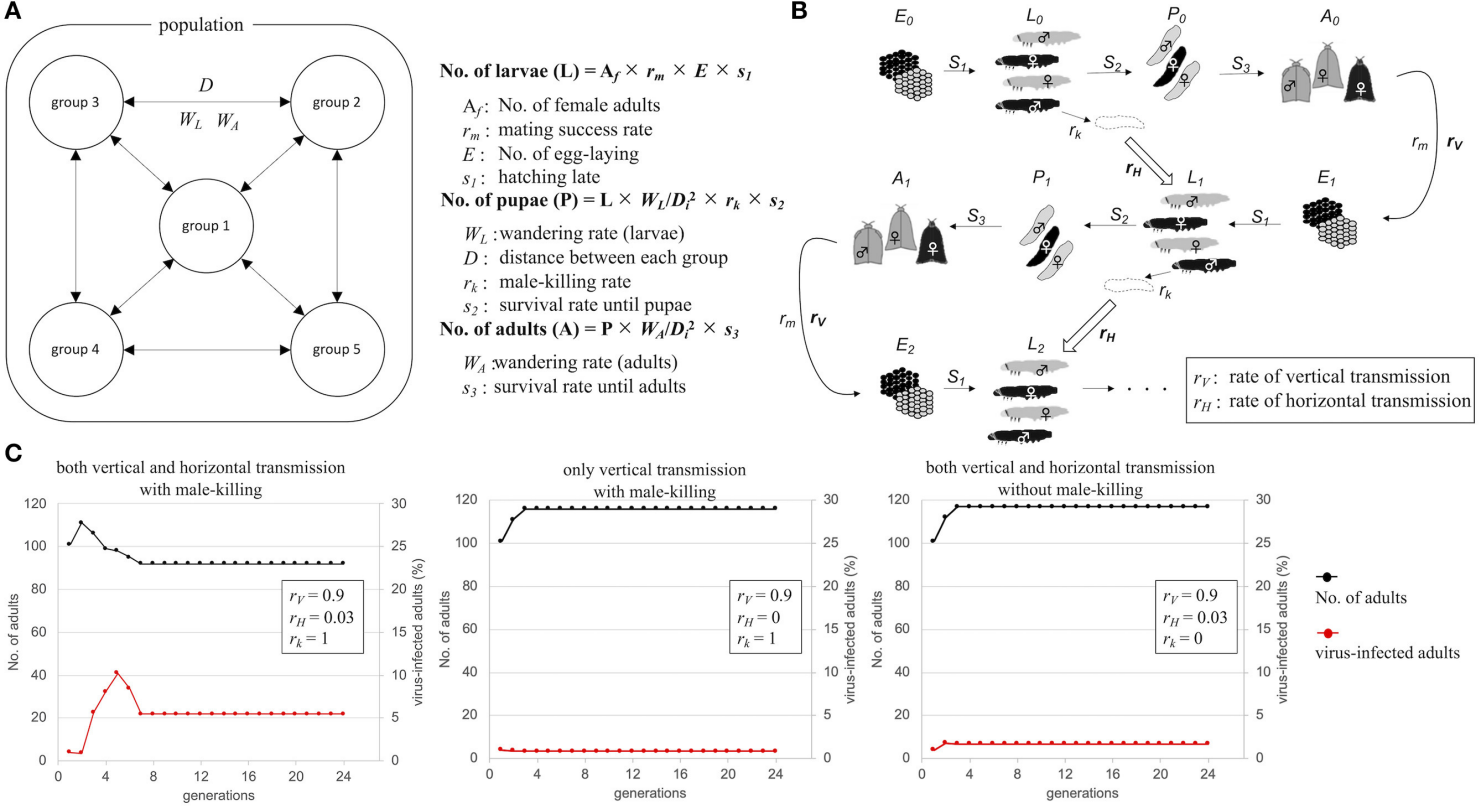
Schematic model of Osugoroshi virus (OGV) infection in a *Homona magnanima* population. **(A)** Population setting for mathematical modeling. An *H. magnanima* population is composed of five separated groups. Some parts of larvae (*W*_*L*_) and adult (*W*_*A*_) moths wander across each group according to the relative distance to another group (*D*). **(B)** Infection model of OGV and through *H. magnanima* populations. During the stages, OGV-infected male larvae (black) go to die (*r*_*k*_) and spread virus progenies, and the larvae in the next generation uptake viruses with a certain probability (*r*_*H*_). OGVs are also transmitted to the next generation from virus-infected females (*r*_*V*_). **(C)** Population simulation of *H. magnanima* after OGV-contamination with various situations. The populations start with 100 naïve individuals and 1 OGV-infected female (black lines). OGV-infected moths (red lines) are maintained throught generations by vertical and/or horizontal transfer.

OGVs were thought to be mainly maintained in female *H. magnanima* through vertical transmission, which implies that the OGV RNAs behave as a maternal genetic factor. If the infection of OGVs does not contribute to the fitness of infected females, the infection rate of OGVs in the population will decrease through generations without the contribution of genetic drift. Indeed, the larval development time of OGV-infected females was prolonged, with a decreased pupal body weight (Takamatsu et al., 2020). Therefore, a propagation strategy other than vertical transmission seems to be required to maintain an OGV population. In the late male-killing, OGVs also multiply in males at least until the larval stage, and then they are spread around the dead males. The progenies of OGVs from the dead males would infect other larvae through horizontal transmission [possibly oral infection, observed in experimental condition (Takamatsu et al., 2020)]. Here we hypothesized that the late male-killing phenotype increases the infection rate of OGVs by horizontal transmission. We built a mathematical model for viral infection rates and host moth numbers through generations (Figure 4A). The model started from 5 populations with 20 adult individuals in each population, where an OGV-infected female was introduced into one of these populations. Larvae and adults interact among the populations according to the distance between each population (*D*) and the wandering of larvae (*W*_*L*_) and adults (*W*_*A*_) (Figure 4B). This model included the oviposition numbers (*E*) and survival rates to the larval stage (*S*_1_), to the pupal stage (*S*_2_), and to the adult stage (*S*_3_). The given parameters led to constant moth populations without virus infection. We also put a mating success rate (*r*_*m*_) because male and female numbers would be varied due to male-killing (male-killing rate = *r*_*k*_). In field observation, OGV-infected male larvae died in leaf-rolls, and sometimes leaf-rolls were reused by larvae in the next generations. Therefore, we considered that the horizontal transmission would occur in the leaf-roll (Figure 4B, blocked arrows). The parameter for vertical transmission rate (*r*_*V*_) was constant here (=0.9). When we put the parameter of the horizontal transmission rate (*r*_*H*_) as 0.03 (Figure 4C, left), the viral infection rate increased in the first four generations (to 10.2%), then decreased and became constant as 5.4%. If the horizontal transmission triggered by late male-killing did not appear (*r*_*k*_ = 0), the infection rate was always constant, like the vertical transmission only model (Figure 4C center and right). Note that the good contribution of the horizontal transmission for viral survival was achieved with a combination of certain population size and horizontal transmission rate. For example, when the model started with twice the population size, the number of virus-infected moths rapidly increased, and the moth population was disrupted by eliminating all-male moths (Supplementary Figure 7, left). The lower *r*_*H*_ gave a moderate increase of viruses in populations without disruption of the moth population even with a higher number of individuals (Supplementary Figure 7, right). Thus, the horizontal transmission via late male-killing could facilitate OGV's spread in *H. magnanima* populations without disrupting the host ecosystem.

## Discussion

The present study is the first report of the isolation of partitivirus from insects, in which we identified the late male-killing virus, OGV. However, two critical issues have yet to be addressed. The first is the actual genomic composition of the late male-killing virus. As described above, we identified 27 partiti-like viral segments, including three segments encoding RdRp. Unfortunately, we are yet to confirm whether all of these segments were coordinated in virus replication and late male-killing in *H. magnanima*. The extracted virion-inoculation experiment showed that late male-killing occured without OGV1 RNA1 (G_1_ strain 16 in Figure 3). This would imply that if the RdRp encoded in OGV1 RNA1 is responsible for the replication of the 23 OGV-related RNAs (Exception being RNA 16; Table 2), then all 23 RNAs will have no role in male killing. In this scenario, OGV2 RNA1, OGV3 RNA1, or OGV-related RNA16 is responsible for the late male-killing phenotype, and OGV2 RNA1-encoded RdRp transcribes OGV-related RNA16 because they share terminal sequences. However, the detection of OGV-related RNA7 in G1 in the absence of OGV1 RNA1, makes this scenario unlikely. It suggests that the recognition of OGV-related RNA segments is not restricted to one RdRp or the other, but shared. Moreover, the fact that partitiviral virions encapsidate each segment singularly makes it difficult to define the actual genomic composition of OGVs (Nibert et al., 2013). To address this issue, some experiments, e.g., a reconstruction of OGV with infectious clone RNA, will be required.

The second issue, regarding the mechanisms of late male-killing by OGVs, is related to the first. OGV infection did not affect hatchability but did induce male-specific toxicity, especially in the larval stage (Supplementary Figure 2). Although the mechanism of late-male killing remains largely unknown, one possibility is a putative toxic gene in OGVs, which functions only in *H. magnanima* males after hatching.

What is the role of male-killing for OGVs? One possible effect is inbreeding inhibition of host *H. magnanima*. Late male-killing results in eliminating males in an egg mass, which would prevent inbreeding among the females. Our field investigation demonstrated that OGVs were widely distributed in Japan and are frequently found in tea fields (Table 1). This observation implies that OGV infection in *H. magnanima* possibly leads to a higher genetic diversity of *H. magnanima* populations.

Viruses require host cells for their replications. Therefore, the good survival of the infected host is crucial for viruses. On the other hand, the viruses' pathogenicity helps virion escape from their host through the disruption of host cells. OGVs kill male *H. magnanima* after hatching. Unlike embryonic male-killing, the late male-killing phenotype would facilitate efficient viral replication according to the increase of host biomass. We hypothesized that late male-killing facilitates the horizontal transmission of OGVs in the *H. magnanima* population, and our mathematical model shows a positive effect on the OGVs population by late male-killing (horizontal transfer) without disruption of host populations and resulted in an equilibrium of infection (Figure 4). The observed percentages of OGV-related RNA7-positive moth in the field study varied between 0 and 41.9% (the average was 13.9%) (Table 1). Our simple model showed the equilibrium infection rate of 5.8%, which was almost consistent with the field observation.

Interestingly, the ratio of virus-infected moth in the “non-male killing virus” is lower than the male-killing model. It indicates the importance of horizontal transfer for OGVs survival. Our model also showed that the rapid propagation of viruses could lead to the disruption of colonies due to the elimination of males (Supplementary Figure 7). The individually packaged virions of partitiviridae RNA segments however reduce the horizontal transmission efficiency of OGV, preventing fatal propagation of OGVs in *H. magnanima* populations.

As we assumed in the mathematical model, the effect of male-killing (elimination of males) in the field would be masked by transmigration of moths, resulting in the observed typical sex ratio (Table 1). This is likely because if there was no masking effect on male-killing, the population would be lost.

*Partitiviridae* has been known as a group of fungal viruses. In this study, we successfully identified and isolated OGVs as *Partitiviridae*-related viruses. As found in Figure 1, the recent metagenomic analysis demonstrated that many invertebrates possess virus-like sequences related to OGVs, suggesting various unidentified potential male-killing viruses in insects.

## Data Availability Statement

The datasets presented in this study can be found in online repositories. The names of the repository/repositories and accession number(s) can be found in the article/Supplementary Material.

## Author Contributions

RF identified OGVs, purified virions, carried out TEM analysis, analyzed viral sequences, wrote the manuscript, and supervised the study. MI planned the experiments, carried out the field surveys and the RACE sequencing, and supervised the study. TT carried the PCR in the diagnosis of bacterial infections. HA and MNi carried out the inoculation experiments. NA examined rates of hatching and fatality of the colony strains. KI supported NGS analysis. MNa carried out the field surveys and supervised the study. SU and YC carried out the FLDS analysis. MA-B supported manuscript preparation with English proofreading. YK conceived the original idea and supervised the study. All authors contributed to the article and approved the submitted version.

## Conflict of Interest

The authors declare that the research was conducted in the absence of any commercial or financial relationships that could be construed as a potential conflict of interest.
