# Supplementary material for: Late Male-Killing Viruses in Homona magnanima Identified as Osugoroshi Viruses, Novel Members of Partitiviridae
Source: Front Microbiol. 2021 Jan 20;11:620623. doi: 10.3389/fmicb.2020.620623 (PMC7854922; doi:10.3389/fmicb.2020.620623)
Supplement: Supplementary file 1 [file Data_Sheet_1.pdf]

Supplementary Table 1 Results of next-generation sequencing analysis using SPAdes assembler algorithm for *Homona magnanima* late strain

Contigs	Length	coverage	*BLASTX or BLASTN	identities
MKsp1	2,739	1.095	Expression vector pKLD66nCBP, complete sequence	2694/2705, n
MKsp2	1,523	7.754	RdRp [Hubei partiti-like virus 33]	334/476, x
MKsp3	1,351	26.196	RdRp [Hubei coleoptera virus 5]	229/430, x
MKsp4	1,351	67.159	Hypothetical protein A2Y79_00980 [Deltaproteobacteria bacterium RBG_13_43_22]	26/88, x
MKsp5	1,311	41.476	N.D.	
MKsp6	1,288	71.196	N.D.	
MKsp7	1,277	118.105	N.D.	
MKsp8	1,274	62.695	Hypothetical protein A2W35_11610 [Chloroflexi bacterium RBG_16_57_11]	20/50, x
MKsp9	1,271	99.761	N.D.	
MKsp10	1,262	36.002	N.D.	
MKsp11	1,260	32.300	GTP cyclohydrolase I FolE [Cobetia crustatorum]	31/110, x
MKsp12	1,259	8.192	N.D.	
MKsp13	1,256	5.477	N.D.	
MKsp14	1,254	13.137	N.D.	
MKsp15	1,251	42.246	Multicopper oxidase [Citrobacter sp. 50677481]	45/183, x
MKsp16	1,247	21.411	N.D.	
MKsp17	1,244	85.232	N.D.	
MKsp18	1,231	43.167	N.D.	
MKsp19	1,225	116.449	Homona magnanima MK1068 RNA for hypothetical protein, partial cds	996/1055, n
MKsp20	1,218	15.753	N.D.	
MKsp21	1,212	14.323	N.D.	
MKsp22	1,204	22.561	N.D.	
MKsp23	1,199	22.424	LysR family transcriptional regulator [Rhizobium mesoamericanum]	19/61, x
MKsp24	1,197	13.975	N.D.	
MKsp25	1,173	13.715	N.D.	
MKsp26	1,169	35.216	N.D.	
MKsp27	1,065	0.929	Enterobacteria phage RB59, complete genome	685/687, n
MKsp28	1,059	57.853	Hypothetical protein [Rheinheimera sp. EpRS3]	27/116, x
MKsp29	995	58.825	Hypothetical protein HMPREF1002_03578 [Porphyromonas sp. 31_2]	32/96, x
MKsp30	986	44.906	RdRp [Hubei coleoptera virus 4]	163/326, x
MKsp31	971	18.769	RdRp [Hubei coleoptera virus 4]	150/300 x

*Each contig was determined in BLASTX and BLASTN, and the top hit with longer coverage was listed. n: identities in BLASTN, x: identities in BLASTX, N.D. = not detected

Supplementary Table 2 Viruses and their NCBI accession numbers used in the phylogenetic analysis

Virus	Genus	NCBI_Accession
Verticillium dahliae partitivirus 1	unclassified	AIN34928.1
Alternaria alternata partitivirus 1	unclassified	APT70074.1
Penicillium aurantiogriseum partitivirus 1	unclassified	ALO50132.1
Soybean leaf-associated partitivirus 2	unclassified	ALM62248.1
Rhizoctonia solani virus 717	Betapartitivirus	AJE29743.1
Pseudogymnoascus destructans partitivirus-pa	unclassified	APG38313.1
Sophora japonica powdery mildew-associated partitivirus	unclassified	AOF47284.1
Verticillium albo-atrum partitivirus-1	unclassified	AIE47665.1
Primula malacoides virus	Betapartitivirus	ABW82142.1
Aspergillus ochraceous virus	Gammapartitivirus	ABV30676.1
Cannabis cryptic virus	Betapartitivirus	AET80949.1
Ophiostoma partitivirus 1	Gammapartitivirus	CAJ31887.1
Vicia cryptic virus	Alphapartitivirus	YP_272125.1
White clover cryptic virus 1	Alphapartitivirus	AAU14889.1
Rhizoctonia solani dsRNA virus 3	Alphapartitivirus	YP_009329885.1
Atkinsonella hypoxylon partitivirus	Betapartitivirus	NP_604476.1
Penicillium stoloniferum virus S	Gammapartitivirus	CAJ01910.1
Pepper cryptic virus 1	Deltapartitivirus	ALR34990.1
Pepper cryptic virus 2	Deltapartitivirus	BAV93049.1
Fig cryptic virus	Deltapartitivirus	YP_004429259.1
Cryptosporidium parvum virus 1	Cryspovirus	O15926.1
Beihai partiti-like virus 2	unclassified	YP_009333351.1
Xinzhou partiti-like virus 1	unclassified	YP_009329868.1
Hubei partiti-like virus 11	unclassified	YP_009329874.1

Supplementary Table 3 Oligonucleotide sequences used in PCR

target gene	oligonucleotide	Sequences
Homona magnanima β-actin	β-actin-f	5´- AACTGGGATGACATGGAGAAGATCTGGC -3´
	β-actin-r	5´- GAGATCCACATCTGCTGGAAGGTGGACAG -3´
Wolbachia 16S rDNA	Wol16S-f	5´- TTGTAGCCTGCTATGGTATAACT -3´
	Wol16S-r	5´- GAATAGGTATGATTTTCATGT -3´
Spiroplasma ribosomal ITS region	Spiro-f	5´- GCTCAACCCCTAACCGCC -3´
	Spiro-r	5´- GGTAGTCACGTCCTTCATCG -3´
MK1241 RNA	MK1241-f	5´- AAGCGACCCGTTATCATTTG -3´
	MK1241-r	5´- AGACGCTGGAAGCTCGTAAC -3´
OGV1 RdRp (MK-11)	MK11-f	5´- CAGTCCTGGTATCCCATGGC-3´
	MK11-r	5´- CAGCGACAACGGAATCATCG-3´
OGV2 RdRp (MK-25)	MK25-f	5´- CGAAATGCCGCCTTGTATGG-3´
	MK25-r	5´- AACGGAACCACTGGGAACTC-3´
OGV3 RdRp (MKsp30)	MK30-f	5´- ACCTGCTTGGCTAATTCACG-3´
	MK30-r	5´- ATCTCGAGCTCGGTCTTCTG-3´
MK1068 RNA	MK1068-f	5´- ACACCTGAGCGATGTACTGC-3´
	MK1068-r	5´- CGGACTCCACCACCAATGTT-3´
MK-1	MK-1f	5´- ACGTTCCCGTCTTTCTGGAC-3´
	MK-1r	5´- CGCTTACGCTGACATCATGC-3´
MK-2	MK-2f	5´- GAGGGAGCTTAGCGTGTCTC-3´
	MK-2r	5´- GATGCGCAAGAAGGCTTAGC-3´
MK-4	MK-4f	5´- AGACTAGCGAGCACCAAACC-3´
	MK-4r	5´- AATTCGCCACTGAGGTACCC-3´
MK-6	MK-6f	5´- TTGCGTAGGCTCCTCAACTG-3´
	MK-6r	5´- ATCGCAGAGGTTCTGGTTCG-3´
MK-7	MK-7f	5´- CTCGAATGAACGAACGGGGA-3´
	MK-7r	5´- GCGCTTAGGCCGACATCTAA-3´
MK-8	MK-8f	5´- TACAGAACCGCACCGCACA-3´
	MK-8r	5´- CGCGGATCTTCCTAGCCGTA-3´
MK-9	MK-9f	5´- TTCGACGTCTTCTGCGATCC-3´
	MK-9r	5´- AGCGTGGCCTATCATTGGAG-3´
MK-10	MK-10f	5´- CCCAATTCACTTCGCCAACG-3´
	MK-10r	5´- ACACATCTGAGGACGAACGG-3´
MK-12	MK-12f	5´- CTGCAAGATCAAGGCACTGC-3´
	MK-12r	5´- GAATCACATCGGCAAGACGC-3´
MK-13	MK-13f	5´- GTTGAGACGGTTGGCGATTG-3´
	MK-13r	5´- GGTGCAACATACGCGGATTC-3´
MK-15	MK-15f	5´- TCCAGCGTCATCGTATCCAC-3´
	MK-15r	5´- AAGAGACGACGTCGACATCG-3´
MK-16	MK-16f	5´- AAGAGCCTCACCATGTGACG-3´
	MK-16r	5´- ATCCACTGACGTGACTAGCG-3´
MK-17	MK-17f	5´- TGAATGCTCACGACCAGGAC-3´
	MK-17r	5´- TGTAGACGCGGCATCTGATC-3´
MK-18	MK-18f	5´- CCGCTCCAGATCGAGAATCC-3´
	MK-18r	5´- GCCTGCTACTCACTCACCTG-3´
MK-19	MK-19f	5´- CGAGGTGGATCTGGCTGTTC-3´
	MK-19r	5´- TTGAGCTTCGTAAACAGCGC-3´
MK-20	MK-20f	5´- TGCTGAGATGGCAGGTTCAG-3´
	MK-20r	5´- TGGACAGCCGATCCATCATG-3´
MK-21	MK-21f	5´- CCGCTTACCGGATACCTGTC-3´
	MK-21r	5´- ATCTACACGACGGGGAGTCA-3´
MK-22	MK-22f	5´- ACCAGATGGCTGCTGATACG-3´
	MK-22r	5´- GTTCGCTGTGATGTGATGCC-3´
MK-23	MK-23f	5´- GATATCTGGACTCCGCCTGC-3´
	MK-23r	5´- TCTCACGCACCGACAGTAAC-3´
MK-24	MK-24f	5´- AGAATGCATCCCTCCACCAC-3´
	MK-24r	5´- TCCGTACCAGAACGCATGAC-3´
MK-26	MK-26f	5´- GCCACCTCCCTCTTATCCAG-3´
	MK-26r	5´- AACTTCTGGTCATGCGCTTG-3´
MK-27	MK-27f	5´- GTTGGTCACGTTGCACATCC-3´
	MK-27r	5´- AGCGTACGACCAACATGACC-3´

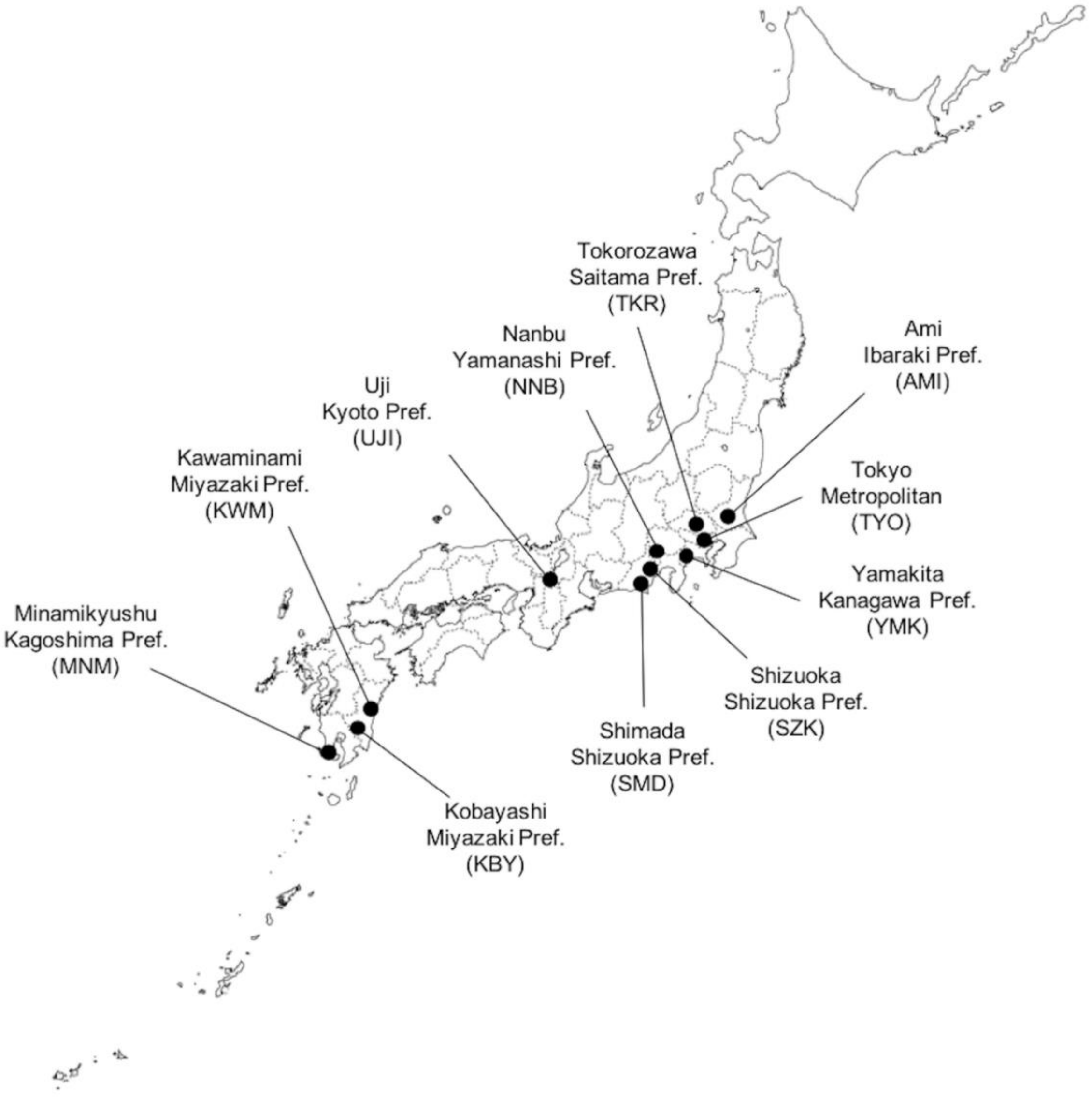

Supplementary Figure 1 Locations of Japanese tea fields where *Homona magnanima* were collected. Abbreviations indicate population names as listed in Supplementary Table 1.

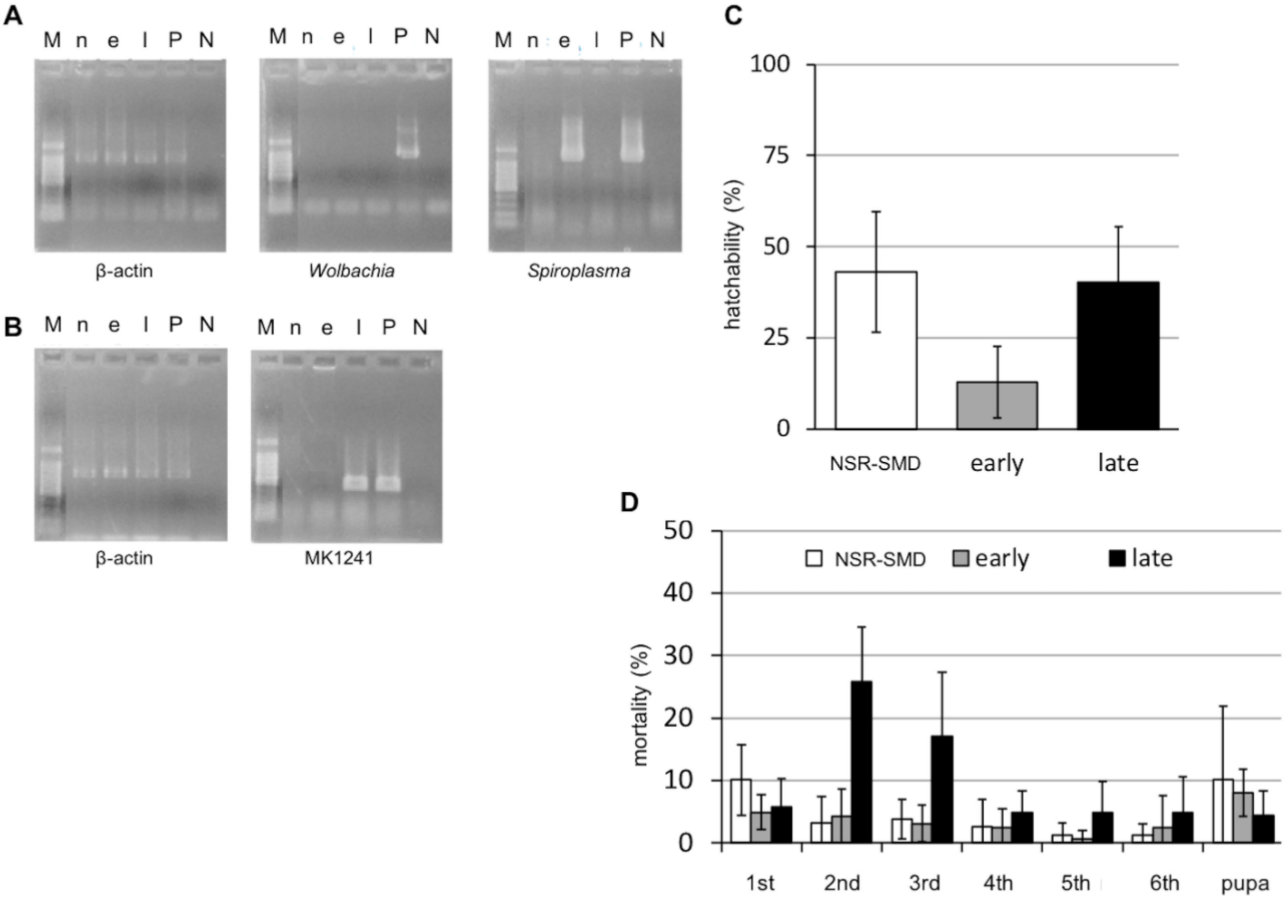

Supplementary Figure 2 Identification of male-killing agents in SMD populations of *Homona magnanima*. **A**: Detection of *Wolbachia* and *Spiroplasma* by PCR. β-actin was chosen as a positive control for DNA extraction. M: size marker, n: non-biased sex ratio strain (NSR-SMD), e: early strain, l: late strain, P: positive control, N: negative control. **B**: Detection of late male-killing virus MK1241 RNA by RT-PCR. β-actin was chosen as a positive control for RNA extraction. The lanes are the same as those in panel A. **C**: Hatchability of laboratory-maintained *H. magnanima* of each strain. The numbers of egg masses were 75 (non-biased sex ratio strain: white bar), 68 (early strain: gray bar), and 51 (black bar). Standard deviations are indicated. **D**: The mortality rate of laboratory-maintained *H. magnanima* of each strain. Mortality was determined at the larval stage and pupal stage. The numbers of neonate larvae used were 159 (NSR-SMD strain: white bars), 163 (early strain: gray bars), and 228 (late strain: black bars). Standard deviations are indicated.

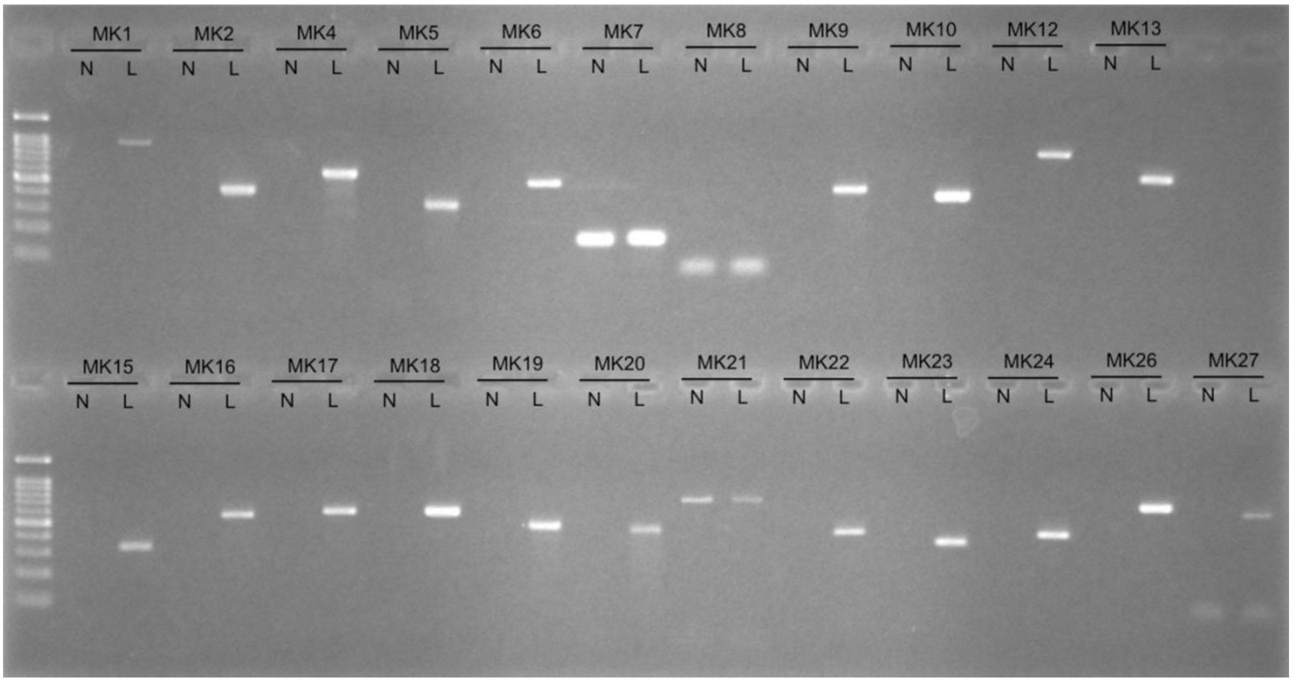

Supplementary Figure 3 RT-PCR analysis for detection of RNAs found in the next-generation sequencing analysis. RT-PCR targeting each contig (listed in Table 1 or Supplementary Table 1) was carried out with RNA isolated from *Homona magnanima* non-biased sex ratio strain (NSR-SMD: N) or late strain (L) adult females.

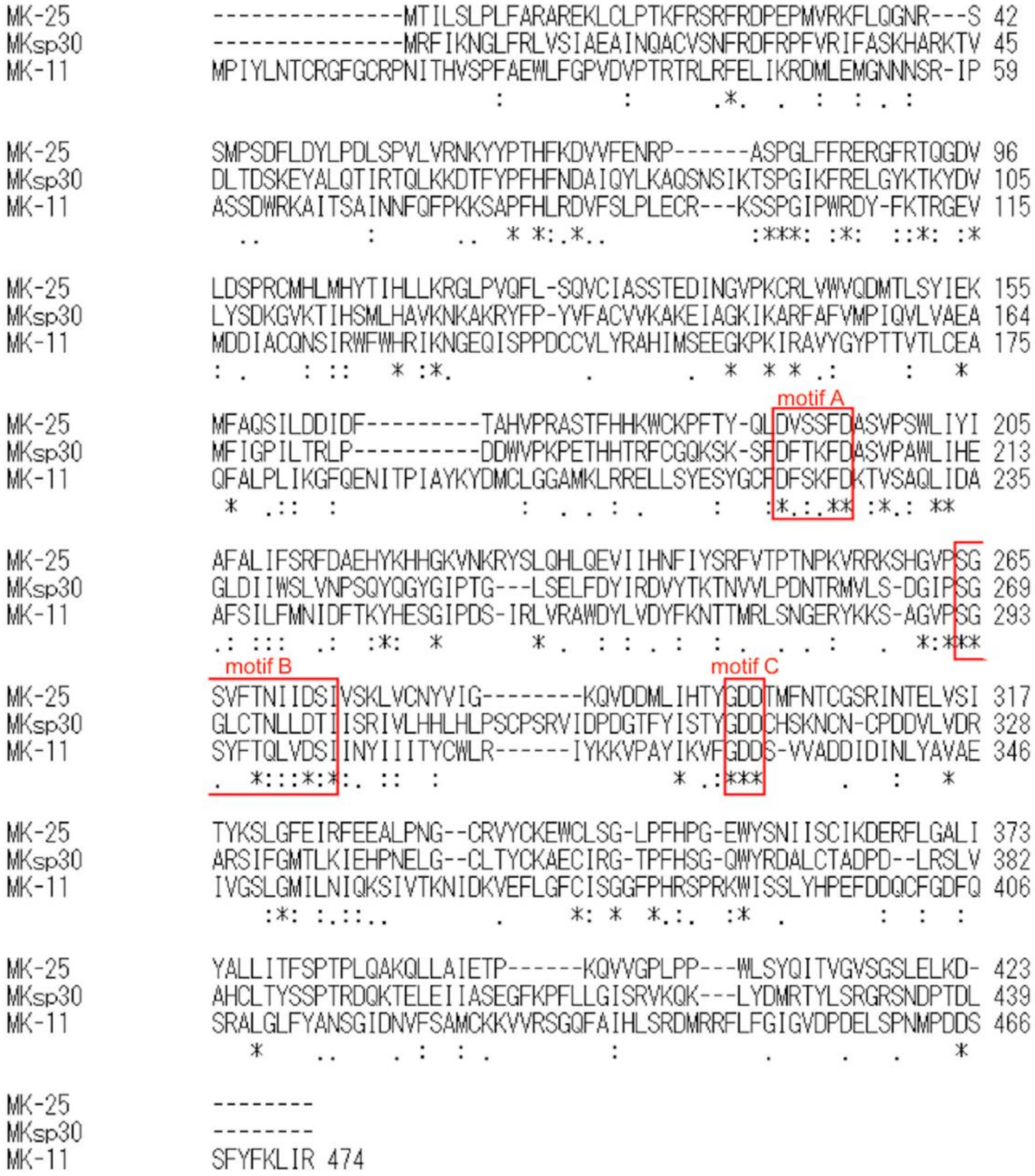

Supplementary Figure 4 Amino acid sequences of partitivirus RdRp found in the *Homona magnanima* late strain and alignment of three identified RdRp genes. The amino acid sequences encoded in the MK-25, MK-11, and MKsp30 contigs were aligned. The double-stranded RNA virus motifs (motif A: DxxxxD, motif B: SGxxxTxxxnsi, motif C: GDD) conserved in RdRp are indicated in red boxes.

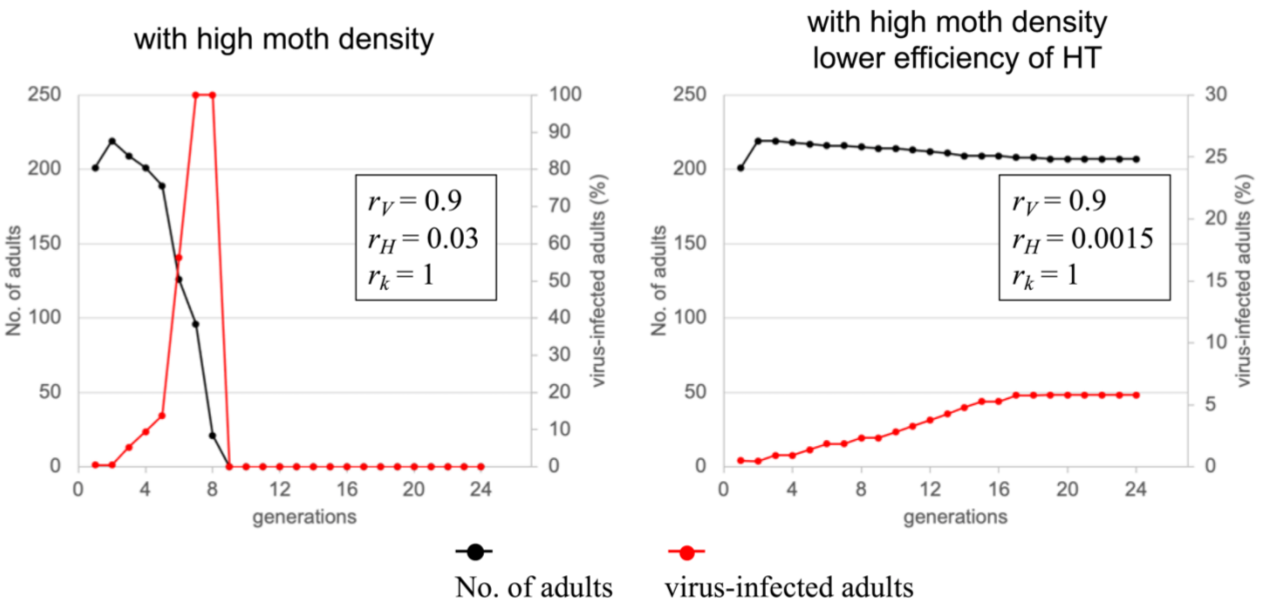

Supplementary Figure 5 Schematic model of Osugoroshi virus (OGV) infection in a *Homona magnanima* population. The left panel shows the expected dynamics of OGVs and host moths with higher moth density than that showed in Figure 4. The right panel showed another dynamics with the same moth density and a lower horizontal transfer rate.
